# A qualitative study on the perception of infection prevention and control measures among healthcare workers without patient contact during the SARS-CoV-2 pandemic

**DOI:** 10.1186/s13756-023-01246-8

**Published:** 2023-04-30

**Authors:** Selina Ehrenzeller, Richard Kuehl, Ana Durovic, Aurélien Emmanuel Martinez, Manuel Battegay, Matthias von Rotz, André Fringer, Sarah Tschudin-Sutter

**Affiliations:** 1 Division of Infectious Diseases and Hospital Epidemiology, University Hospital Basel, University of Basel, Basel, Switzerland; 2 School of Health Professions, Institute of Nursing, Zürich University of Applied Sciences ZHAW, Winterthur, Switzerland; 3 Department of Clinical Research, University Hospital Basel, University of Basel, Basel, Switzerland

**Keywords:** SARS-CoV-2, COVID-19, Infection prevention and control, Personal protective equipment, Safety, Qualitative study, Employee safety, Pandemic management, Interview study, Health care workers

## Abstract

We conducted a qualitative interview-based study to examine the perception of infection prevention and control (IPC) measures implemented during the COVID-19 pandemic among healthcare workers (HCWs) without patient contact in a tertiary academic care center. We compared these findings to those derived from interviews of HCWs with patient contact from the same institution using the same study design. The following main four themes were identified: (1) As for HCWs with patient contact, transparent communication strongly contributes to employees’ sense of security. (2) Information on personal protective equipment (PPE) usage needs to be stratified according to different educational backgrounds and professions. (3) Consistency of IPC measures was positively perceived yet a desire for constant reminders to counteract the fatigue effect played a more significant role for HCWs without patient contact. (4) As compared to HCWs with patient contact, HCWs without patient contact preferred uniform digital training resources rather than more face-to-face training. This study shows that the needs of HCWs with and without patient contact differ and need to be considered in pandemic management.

**Supplementary Information:**

The online version contains supplementary material available at 10.1186/s13756-023-01246-8.

## Background

Preventing transmission of SARS-CoV-2 within healthcare settings has represented one of the major challenges during the COVID-19 pandemic. Most infection prevention and control (IPC) efforts were targeted at healthcare workers (HCWs) with patient contact, thus at the highest risk of exposure to infected patients. Less attention may have been paid to HCWs without patient contact, such as medical technical assistants, laboratory staff, kitchen personnel, cleaning, and administrative staff, all critical in maintaining a hospital’s functionality and at increased risk for exposure to SARS-CoV-2 mainly by contacts among each other. In addition to the physical risk of infection, especially during the early phases of the pandemic, the risk of psychological stress was reported in multiple studies [1–4]. The anxiety of being infected has been shown independently on whether HCWs are directly involved in caring for COVID-19 patients or not [5]. We thus investigated the impact and perception of institutional actions, strategies, and policies aimed at preventing the transmission of SARS-CoV-2 within our institution among HCWs without patient contact. We further compared these results to a prior study conducted at our institution focusing on HCWs with patient contact and applying the same qualitative study design [6].

## Methods

A qualitative descriptive research design [7] using content analysis was performed at the University Hospital Basel, a tertiary care center with more than 40’000 hospital admissions annually. The study was performed in October 2020 just before the deadliest wave of SARS-CoV-2 infections and COVID-19 diseases started in Switzerland in November 2020 [8]. The study was conducted as a follow-up to a study with HCWs with patient contact in the same hospital using the identical methodology to be able to compare differences between HCWs with and without patient contact [6].

We used purposeful sampling [9] to contact the HCWs by email from ten different departments including radiology, pathology, occupational health department, human resources, communications, hospital canteen, hospital safety department, cleaning service, patient administration, and patient bed allocation services. The participants represented departments and divisions essential to maintaining the hospital’s functionality but had no direct patient contact, defined as not providing hands-on care to patients. We conducted fourteen (n = 14) semi-structured interviews which on average lasted 27 min. The interview guide used had been developed for the study with HCWs with patient contact and had been created based on a literature review as well as the results of a peer debriefing with IPC specialists from the hospital. Of the interviewed HCWs, 11 held senior and 3 held non-senior positions within their departments. A more detailed description of the characteristics of participants is provided in the Additional file 1, Supplementary Table 1.

Using a qualitative descriptive research design [7, 10], we performed a deductive-inductive content analysis of the transcribed interview content according to Schreier [11]. For more detailed information on the methodology please refer to the Additional file 1, Supplementary file 1, and for the Interview Guide Additional file 1, Supplementary file 2. The main categories that resulted from the study with HCWs with patient contact [6] were derived from the interview guide. These main categories were used as the underlying concept upon which the analysis for this article was deductively built upon. New categories were then created that emerged inductively from the text. We adhered to the consolidated criteria for reporting qualitative studies (COREQ) which can be found in Additional file 1, Supplementary Table 2 [12].

Quotations illustrating the main themes were translated from German to English with minimal adjustments so as not to lose the meaning of the quotation. The quotations are followed by the interview number, e.g., “A3”. The allocation to position and department can be seen in Additional file 1, Supplementary Table 1. The ethics committee confirmed that approval was not required (EKNZ-Request-2020-00931) as this is a quality assessment project. Nevertheless, all HCWs provided written informed consent.

## Findings

We identified four main themes that emerged as significant concerning pandemic management and perceptions of HCWs without patient contact. Themes and subthemes are summarized in Table 1. The main themes were mentioned by all 14 interviewees (100%). The subthemes emerged inductively as the most frequently mentioned topics.

**Table 1:** Main themes related to safety perception identified through the interviews and mentioned by 100% of participants, as well as sub-themes that emerged inductively from the main themes

**Transparency and clarity of information**
– Advantage of contact persons responsible for individual departments
– Need for clear responsibilities and wish for involvement
– Necessity for medial interpretation of media reports & hospitalizations
**Instructions on the use and availability of PPE**
– Information needs to be stratified according to different educational backgrounds and professions
**Consistency of IPC measures**
– Positive perception of area closure & visitor regulations
– Desire for constant reminders to counteract the fatigue effect
**Resources and Teaching**
– Recognition of the need for pathogen unspecific pandemic concepts
– Support through uniform digital resources & functioning IT structures

## Transparency and clarity of information

### Advantage of direct contact persons responsible for individual departments

In departments that had direct contact persons within the division of infectious diseases and hospital epidemiology to pass on information, this was mentioned as extremely helpful and essential for their sense of security, e.g., as was established for the radiology and later also the pathology department. In contrast, in departments without direct and constant contact persons, information regarding IPC measures from the institution was often perceived as confusing and its receipt as time-consuming.

### Need for clear responsibilities and wish for involvement

Departments directly involved in receiving visitors to the hospital or directly involved in diagnostic procedures such as the laboratory or radiology, frequently mentioned that decisions relevant to them were made without their involvement, and which were thus not logistically well thought out and responsibilities were not clearly allocated. Further, they were informed about such decisions with delay. This resulted in frustration and the wish to be represented within the decision-makers in the hospital during the pandemic.

### Necessity for medical interpretation of media reports & hospitalizations

Difficulties in interpreting communications from the hospital management, e.g., regarding the current number of hospitalized patients, etc. but also public media reports in general, were mentioned several times. As a large part of the staff in the departments interviewed did not have a medical background, this may have led to increased uncertainty. Brief comments from medical experts within the institution on the current epidemiological situation were perceived to be more helpful than simply providing a figure.

## Instructions on the use and availability of personal protective equipment

### Information needs to be stratified according to different educational backgrounds and professions

Knowledge of the “correct” type of protective mask was a concern for staff from all departments. As face mask supplies were limited, the number of masks to be used was restricted to one per week for each employee, unless visibly soiled. This led to resentment among HCWs from different departments as they highlighted different exposures, e.g., fumes and humidity in the canteen kitchen, which made it impossible to wear the same mask for several hours. As a senior kitchen employee explains: “*Face masks are a bit special because they* [kitchen staff] *work in a relatively warm area exposed to humidity and steam and we had, and we still have, a bit of a struggle to get people to wear the face masks properly. That was the main challenge.” (A4).*

Several times it was mentioned that adapted information should be available for individual departments with different requirements (e.g., surgical masks for HCWs without patient contact, but FFP2 during patient contact) and that this should also be adapted in teaching materials such as videos. Training videos for example were recorded at the beginning of the pandemic recommending the use of FFP2 masks for the cleaning service when cleaning/disinfecting rooms of patients with COVID-19. Throughout the pandemic, surgical masks were recommended, but the training videos were not adapted. A cleaning employee reports: *“I was quite afraid when I saw such seriously ill people. How does the mask protect us? (…) Afterward, I think I said: “This is my job. I have to do this.“ And now it’s like normal for me when I go into the room.“ (A3).*

## Consistency of IPC measures

### Positive perception of area closure & visitor regulations

In the initial stages of the pandemic, most interviewees supported the strict restrictions such as premises lockdown and visitor ban. Opinions differed on the details, e.g., whether the military should be responsible for monitoring the entrances to the hospital and whether the enforcement was sufficient or not. *“I think you must adapt* it [i.e., areal closure and visitor regulations] *to the phase of the crisis: who secures the access, how you secure it, and on the site: whom you employ where. Which soft skills are needed where, and how to deal with people.” (A8)*. Several HCWs mentioned that there should be exceptions to the visitor ban and that these should ideally be predefined so that the decision does not have to be made by an individual, e.g., permission for a visit shortly before the end of a patient’s life.

### Desire for constant reminders to counteract the fatigue effect

HCWs without patient contact reported difficulties getting used to the new IPC regulations, especially at the beginning of the pandemic. Several HCWs explained that repeated reminders in the form of disinfection dispensers, posters, and mask dispensers helped make the new measures a habit. *“You must stumble over a hand rub dispenser every two meters; you must see a sign on every wall: “Put on your mask!“ (A8).* And as another HCW put it: *“And at the beginning (…) you may have forgotten. But because so many hand rub dispensers, for example, were set up, you could walk somewhere and think: True*! [I should] *wash my hands, disinfect my hands. That becomes like an automatic thing. Just like wearing the mask.” (A5).*

## Resources and teaching

### Recognition of the need for pathogen unspecific pandemic concepts

Across all interviewed departments, the opinion was that a general “pandemic concept” already exists or should exist to better manage the initial phase of a pandemic. Only the hospital canteen had such a concept ready and was thus able to switch to a “pandemic mode” from one day to the next. This led to a strong sense of “feeling prepared” within the team with a concept ready to use and less stress as experienced by HCWs from other departments without prewritten concepts. Interviewees recognized differences in department-specific needs regarding IPC measures, as well as challenges predicting the trajectory of the pandemic. Pathogen-unspecific concepts may thus be of limited utility, as pointed out: *“In the end, I think the crucial thing is people’s flexibility and then being able to react to situations”. (A14).*

### Support through uniform digital resources & functioning IT (information technology) structures

The diversity of the different departments led to inhomogeneous needs for training and support. However, the desire for more uniform digital training resources (such as videos and pictures) concerning PPE was present across several departments. *“A video (…) says more than a thousand pages of text (…). Whether it is the correct ventilation in the prone position of a patient, the correct performance of a posterior nasal swab, or even the correct handling of a medical or FFP2 mask, which you put on and then dispose of again.” (A10)*. The importance of well-functioning IT structures within a pandemic was also mentioned frequently. Difficulties with essential data transfer at the start of the pandemic complicated work across several departments.

## Discussion

Our findings show the most important factors influencing the perception of safety among HCWs without patient contact during a pandemic. The identical methodology as a previously conducted study with HCWs with patient contact [6] allows a direct comparison with the findings influencing the perception of safety and showing their concerns. See Table 2 for a tabular comparison of the main themes.

**Table 2:** Direct comparison of main categories among HCWs with and without patient contact

HCWs without patient contact	HCWs with patient contact
Transparency and clarity of information	
Instructions on the use and availability of PPE	Material availability (Shortage, Alternatives)
Uniformity and consistency of guidelines & IPC measures	
Preferring uniform digital resources for training	Preferring personal face-to-face teaching
	Support and appreciation of personnel

Our findings suggest that there are major similarities but also differences between departments and different occupational groups. In both groups, transparent communication and clear information were the most important aspects mentioned. Regarding sub-themes, HCWs without patient contact commonly felt “left out” and not included, even though the decisions taken ultimately had a strong influence on their daily work. Likewise, the interviewees without medical training showed a lack of understanding of necessary IPC measures and PPE, as well as a desire to be informed by medical professionals about current events in the hospital. The only main theme that was mentioned by HCWs with patient contact but not by HCWs without patient contact was the aspect of support and appreciation. HCWs with patient contact mentioned for example personal support including time for discussion of difficult situations with their superior, easy access to the hospital`s testing facility, and general acknowledgment of their work as important aspects, which did not seem to be a main theme for HCWs without patient contact. Nevertheless, it is important to mention that compliance among HCWs without patient contact is crucial for functioning pandemic management and therefore appreciation and acknowledgment of their work is also important.

Perception of PPE shortage differed between HCWs with and without patient contact. For HCWs with patient contact, the lack of availability of PPE, mainly masks, was a burden. HCWs without patient contact were, in contrast, mainly troubled by regulations regarding the minimum wearing time (e.g., in the kitchen) and the lack of knowledge on correct use. In contrast to the HCWs with patient contact, most staff without patient contact primarily did not express the need for face-to-face training but rather required easily accessible and simple videos and images. This is to support HCWs with different levels of language skills and educational backgrounds. The issue of providing consistent rules and their implementation challenged HCWs from all departments – yet in different ways. In contrast to the HCWs with patient contact, HCWs without patient contact mentioned the need for constant reminders and assistance to comply with the IPC measures.

Our findings are reinforced by other reports. Adeyemo et al. similarly pointed to effective communication and transparency as important factors in leading HCWs during a crisis. The majority of HCWs surveyed were HCWs with patient contact, but 8.9% had not yet cared for a COVID patient [13]. The qualitative study by Jeleff et al. also included two HCWs without patient contact [14]. The problem of shortages of PPE, and lack of preparedness including delayed IPC guidelines and lack of acknowledgment (especially mentioned by nurses) were important findings that support ours. Our study analyzed the group of HCWs without patient contact separately. To our knowledge, no other study has been conducted exclusively with HCWs without patient contact. A review article in 2022 mentioned the need for studies with “other HCWs than doctors and nurses” [15]. Our study fills this gap.

Our study has some important limitations. A generalization of the findings is only possible to a limited extent due to the monocentric and qualitative study design. To ensure objectivity, an early career researcher and medical doctorate candidate (SE) conducted the interviews without being familiar with the HCWs beforehand. It should also be noted that recall bias may have occurred, as the interviews were conducted several months after the initial phase of the pandemic in Switzerland and 1–2 months later than the interviews with the HCWs with patient contact. Another limitation is the fact that only three (n = 3) non-senior and eleven (n = 11) senior HCWs were interviewed, which is why comparisons between senior vs. non-senior were not performed. Few interviewees had brief encounters with COVID-19 patients, but not to the same extent as physicians and nurses directly involved in caring for infected patients. These encounters included, the performance of autopsies cleaning and disinfecting the rooms of COVID-19 patients or being involved in repositioning of patients prior to the performance of CT scans.

## Conclusion

Our study identifies institutional regulations and measures which may have influenced HCWs’ perception of safety during the COVID-19 pandemic. Among them, clear and direct communication is one key component in pandemic management. HCWs not directly involved in patient care and thus often excluded from direct decision-making may need individualized approaches to ensure adequate information and to improve their perception of safety. The needs of HCWs with and without patient contact differ and need to be considered to improve pandemic management.

## Electronic supplementary material

Below is the link to the electronic supplementary material.

**Supplementary Material 1: Supplementary Table 1.** Characteristics of participants. **Supplementary File 1.** Methods. **Supplementary File 2.** Interview Guide. **Supplementary Table 2.** COREQ (Consolidated criteria for reporting qualitative studies): 32-item checklist

## Data Availability

The dataset generated and analyzed during the current study is not publicly available due to individual privacy possibly being compromised but is available from the corresponding author on reasonable request.

## List of abbreviations

COVID-19: Coronavirus disease of 2019
EKNZ: Ethics Commission of Northwestern and Central Switzerland
HCWs: Healthcare workers
IPC: Infection prevention and control
IT: information technology
PPE: Personal protective equipment
SARS-CoV-2: severe acute respiratory syndrome coronavirus 2
