# Supplementary material for: A qualitative study on the perception of infection prevention and control measures among healthcare workers without patient contact during the SARS-CoV-2 pandemic
Source: Antimicrob Resist Infect Control. 2023 Apr 30;12:43. doi: 10.1186/s13756-023-01246-8 (PMC10149153; doi:10.1186/s13756-023-01246-8)
Supplement: Supplementary file 1 — Supplementary Material 1: Supplementary Table 1. Characteristics of participants. Supplementary File 1. Methods. Supplementary File 2. Interview Guide. Supplementary Table 2. COREQ (Consolidated criteria for reporting qualitative studies): 32-item checklist [file 13756_2023_1246_MOESM1_ESM.docx]

*Additional file 1*

**Supplementary Table 1. Characteristics of participants**

| *Nr.* | *Department* | *Profession* | *Position* |
| --- | --- | --- | --- |
| A1 | Patient Bed Allocation Service | Head of Patient Bed Allocation Service (for 6 years) | Senior |
| A2 | Patient Administration | Head of Patient Administration (for >10 years) | Senior |
| A3 | Cleaning service | Cleaner (for >20 years) | Non-Senior |
| A4 | Hospital canteen | Head of Kitchen (for >9 years) | Senior |
| A5 | Hospital canteen | Chef (for 6 years) | Non-Senior |
| A6 | Cleaning service | Head of Cleaning service (for 5 years) | Senior |
| A7 | Occupational Health Department | Staff Physician (for 4 years) | Senior |
| A8 | Communications | Media spokesman (for 1 year) | Senior |
| A9 | Human Resources | Head of Human Resources (for 2 years) | Senior |
| A10 | Pathology | Physician (for 10 years) | Senior |
| A11 | Radiology | Hygiene Expert, Radiology | Non-Senior |
| A12 | Radiology | Nurse in charge (for 12 years) | Senior |
| A13 | Hospital Safety Department | Head of Security (for 5 years) | Senior |
| A14 | Radiology | Physician (for 11 years) | Senior |

**Supplementary File 1***.* **Methods**As part of our study, fourteen (n = 14) semi-structured interviews were conducted with employees of a tertiary care hospital to identify institutional policies and actions that affected employees' perceptions of safety during the first wave of the COVID‐19 pandemic. Its methodology was identical to that of the study of frontline HCWs, allowing direct comparison and discussion of the results. (1)

**Setting**At University Hospital Basel (USB), a tertiary care hospital with over 40'000 admissions per year and 813 beds, we conducted a qualitative interview study. Located in Basel (approximate population: 200,000), it serves as a referral center for patients requiring specialized medical care in the northwestern part of Switzerland. At the beginning of the pandemic, a task force was set up to determine the pandemic management at the hospital. A task force led by hospital management met one to three times a week, with representatives from different departments.

**Participants**

To choose the participants for the interviews a purposeful and criterion-based sampling was performed to include hospital employees who were involved in patient flow during the initial acute phase of the SARS-CoV-2 pandemic in March 2020 but had no patient contact.
We used email to contact HCWs (n=14) from ten different departments including radiology, pathology, occupational health department, human resources, communications, hospital canteen, hospital safety department, cleaning service, patient administration, and patient bed allocation services. All departments had a crucial involvement through administrative work or diagnostics relevant to COVID-19 patients but were not involved in frontline patient care at the bedside. Of the interviewed HCWs 11 held senior and 3 held non-senior positions within their departments. A more detailed description of the characteristics of participants is provided in Additional file 1, Table 1.

**Data collection**

Face-to-face semistructured interviews were conducted by a medical doctoral candidate (SE) in October 2020. She was not involved in developing the measures that were taken in the hospital. When obtaining informed consent, she explained the study goal and interview procedure to participants and then repeated them in her opening remarks before the interview.
The semi-structured interview guide used had previously been used in interviewing HCWs in the same hospital with patient contact during the pandemic and had been developed based on the objectives and personal experiences with the COVID-19 situation from the perspective of the division of infectious diseases & hospital epidemiology. Due to the lack of significance for HCW without patient contact, the interviewees were not asked about scientific activity and diagnostics within the hospital and this was adjusted in the interview guide at the beginning. The interview guide consists of seven topics that were discussed in a peer debriefing to have been the most important and which were used as a coding frame for data analysis: (1) material availability, (2) IPC guidelines, (3) training opportunities, (4) security (including visitor management), (5) communication, (6) data management, and (7) preparedness and personal lessons learned. The modified interview guide can be found in Additional file 1, Supplementary file 2. We digitally recorded and transcribed verbatim all interviews and excluded personal identifiers.

**Data analysis**

Using a qualitative descriptive research design (2), we performed a deductive-inductive content analysis of the transcribed interview content according to Schreier (3). In a clear step-by-step process, initial categories were formed by categorizing the transcript statements and using the main themes that resulted from the frontline HCWs study (1) regarding “security factors” among HCWs as the underlying concept upon which the analysis was deductively built. Secondly, in an inductive manner, new categories were then created within the main themes that emerged from the text and formed the subthemes. An analysis was then conducted based on categories and compared between departments for each category.
An external consultant provided feedback on the coding and methodology we used to ensure the accuracy of the methodology. We adhered to the Consolidated criteria for reporting qualitative studies (COREQ) (4), see Additional file 1, table 2. All data were managed and analyzed with MAXQDA 2020 (VERBI Software GmbH, Berlin) (5).
Numbers have been deliberately omitted in the article and descriptive words such as "most, some, few" are being used instead. Using precise numbers and percentages may be misleading and does not add much value to a qualitative study (6, 7).

**Quality criteria**

Qualitative research quality criteria can be summarized as “trustworthiness” (8). To be trustworthy, the study must have been conducted fairly and following ethical principles, and the results must be representative of the experiences of the HCWs (9). To strengthen the trustworthiness of this qualitative study (7), we used peer debriefing with experts in medicine and qualitative research. Additionally, we kept an audit trail during the coding and analysis process and present disconfirming evidence.

**References**

1. Ehrenzeller S, Durovic A, Kuehl R, Martinez AE, Bielser M, Battegay M, et al. A qualitative study on safety perception among healthcare workers of a tertiary academic care center during the SARS-CoV-2 pandemic. Antimicrob Resist Infect Control. 2022;11(1):30.

2. Sandelowski M. Whatever happened to qualitative description? Res Nurs Health. 2000;23(4):334-40.

3. Schreier M. Qualitative content analysis in practice: Sage publications; 2012.

4. Tong A, Sainsbury P, Craig J. Consolidated criteria for reporting qualitative research (COREQ): a 32-item checklist for interviews and focus groups. International journal for quality in health care. 2007;19(6):349-57.

5. Kuckartz U. Analyzing Qualitative Data with MAXQDA : Text, Audio, and Video. 1st ed. 2019. ed. Rädiker S, editor. Cham: Springer International Publishing, Imprint: Springer; 2019.

6. Weiss RS. Learning from strangers: The art and method of qualitative interview studies: Simon and Schuster; 1995.

7. Padgett DK. Qualitative methods in social work research: Sage publications; 2016.

8. Lincoln YS, Lincoln YS, Guba EG, Guba EG. Naturalistic inquiry. Beverly Hills [etc: Sage Publications; 1985.

9. Ely M, Anzul M, Freidman T, Garner D, McCormack-Steinmetz A. Doing qualitative research: Circles within circles: Psychology Press; 1991.

**Supplementary File 2. Interview Guide [translated from German to English]**

*Basic questions (related to all topics):*

- What positive and negative aspects can you think of about topic "X"? [What was good/bad?]

- Which aspects would you implement in the same way in the future and which differently?

- What wishes do you have for your department/area in relation to this topic?

*Opening questions*

- First of all, I would like you to describe your function at the USB in the past months and how long you have been working in this position.

- How long have you worked in contact with COVID-19 patients?

- Did you have any previous experience in isolation management and how long was it?

1. **Material** **availability**

[Masks (mouth-nose protection, FFP2), gloves, goggles, over-aprons, hand sanitizer, surface sanitizer, other materials?]

How did you perceive instructions regarding the correct protective equipment?

[Positive/negative, understanding of indication, material (which material should be used when), procedure (sequence, donning and doffing)].

2. **Infection Prevention and Control guidelines (COVID-19 manual)**

[Understanding, availability, completeness, currency/updates]

3. **Training options for the use of protective equipment.**

[Options: Pictorial instructions, videos, on site, walk in, experience with untrained staff (e.g. military, medical students, employees from other hospitals)]

4. **Safety**

[personal infection risk, theft, visitor management, security guards]

5. **Communication**

[internal, external, availability of information, interdisciplinary cooperation],

How did you perceive the collaboration with the Federal Office of Public Health or cantonal medical service, if you had one?

7. **Surveillance (Data Management)**

[Handling of data, overview of infected persons, hospitalized persons, etc.]

8. **Preparation**

1. How well prepared did you feel for a pandemic in your work environment?
2. Did your area/department have a pandemic concept so far or do you know if one is now being planned?
   1. If so, how long has it been in place and could it be used?
   2. If not, what are the arguments against developing such a concept?

9. **Personal Lessons Learnt**

1. What lessons do you draw from the past months in relation to the IPC measures? [more general lessons learned]

10. Are there any other points you would like to add that I have not addressed?

**Supplementary Table 2. COREQ (Consolidated criteria for reporting qualitative studies): 32‐item checklist.** (4)

| **Number & Item** | **Guide questions and description** | **Additional comments** | **Reported on page no. or**  **not applicable (N/A)** |
| --- | --- | --- | --- |
| **Domain 1: Research team and reﬂexivity** | | | |
| *Personal Characteristics* | | | |
| 1. Interviewer/  facilitator | Which author/s conducted the interview or focus group? | SE | Additional file 1.  Supplementary file 2 |
| 2. Credentials | What were the researcher’s credentials? E.g. PhD, MD | cand. med. | N/A |
| 3. Occupation | What was their occupation at the time of the study? | medical student and doctoral candidate who was employed by the University Hospital Basel (USB) during the study period. | N/A |
| 4. Gender | Was the researcher male or female? | female | N/A |
| 5. Experience and training | What experience or training did the researcher have? | SE had no previous experience in qualitative research but deepened her knowledge by literature research and attended online courses to best understand and implement the methodology. | N/A |
| *Relationship with participants* | | | |
| 6. Relationship established | Was a relationship established prior to study commencement? | No. | N/A |
| 7. Participant knowledge of the interviewer | What did the participants know about the researcher? e.g. personal goals, reasons for doing the research | Since SE was not known to the participants, her role and function, as well as the goal and procedure of the interviews, were explained to the participants in the informed consent beforehand and declared again in the opening remarks before the interview began. | N/A |
| 8. Interviewer characteristics | What characteristics were reported about the interviewer/facilitator? e.g. Bias, assumptions, reasons and interests in the research topic | The participants were aware SE was a doctoral candidate and medical student interested in the “Lessons Learnt” of the acute first phase of the SARS-CoV-2 pandemic. | N/A |
| **Domain 2: study design** | | | |
| *Theoretical framework* | | | |
| 9. Methodological orientation and Theory | What methodological orientation was stated to underpin the study? e.g. grounded theory, discourse analysis, ethnography, phenomenology, content analysis | Data were analyzed using content analysis according to Schreier. | Methods, p. 3 |
| *Participant selection* | | | |
| 10. Sampling | How were participants selected? e.g. purposive, convenience, consecutive, snowball | The sampling was purposeful, and criterion based. | Methods, p. 3 |
| 11. Method of approach | How were participants approached? e.g. face-to-face, telephone, mail, email | Contact was made by email. | Methods, p. 3 |
| 12. Sample size | How many participants were in the study? | 14 participants. | Methods, p. 3 |
| 13. Non-participation | How many people refused to participate or dropped out? Reasons? | One employee that was contacted did not participate personally but recommended to another employee that would be more suitable. No one dropped out once agreeing to participate. | N/A |
| *Setting* | | | |
| 14. Setting of data collection | Where was the data collected? e.g. home, clinic, workplace | The interviews were conducted either in the office of the employee, a meeting room in their department or in a private meeting room arranged by the interviewer on hospital campus. | N/A |
| 15. Presence of non-participants | Was anyone else present besides the participants and researchers? | Besides the researcher and participant, no one else was present during the interviews. | N/A |
| 16. Description of sample | What are the important characteristics of the sample? e.g. demographic data, date | Fully presented in Methods section | Methods, p. 3 |
| *Data collection* | | | |
| 17. Interview guide | Were questions, prompts, guides provided by the authors?  Was it pilot tested? | The interview guide is provided as supplementary material (translated from German to English). | Additional file 1.  Supplementary file 2 |
| 18. Repeat interviews | Were repeat interviews carried out? If yes, how many? | No repeat interviews were carried out. | N/A |
| 19. Audio/visual recording | Did the research use audio or visual recording to collect the data? | The interviews were audio-recorded. | Additional file 1.  Supplementary file 1 Methods |
| 20. Field notes | Were ﬁeld notes made during and/or after the inter view or focus group? | SE took field notes during the interview and added to the interview protocol afterwards. These included points such as whether the participant came noticeably prepared with notes or emphasized on individual points particularly. | N/A |
| 21. Duration | What was the duration of the interviews or focus group? | On average, one interview lasted 27:10 minutes (between 14.18 min – 38:24 min). | Findings, p. 8 |
| 22. Data saturation | Was data saturation discussed? | No. | N/A |
| 23. Transcripts returned | Were transcripts returned to participants for comment and/or correction? | The transcripts were not returned. | N/A |
| **Domain 3: analysis and ﬁndings** | | | |
| *Data analysis* | | | |
| 24. Number of data coders | How many data coders coded the data? | The interviewer SE coded the data and discussed her findings with other researchers in the project team. | Methods, p. 3 |
| 25. Description of the coding tree | Did authors provide a description of the coding tree? | No coding tree is provided. | N/A |
| 26. Derivation of themes | Were themes identiﬁed in advance or derived from the data? | The main themes resulted from the study with patient contact were (1) used as the underlying concept upon which the analysis was deductively built. Inductively new categories were then created that emerged inductively from the text | Methods, p. 3 |
| 27. Software | What software, if applicable, was used to manage the data? | MAXQDA | Additional file 1.  Supplementary file 2 Methods |
| 28. Participant checking | Did participants provide feedback on the ﬁndings? | No, a member check was not carried out. | N/A |
| *Reporting* | | | |
| 29. Quotations presented | Were participant quotations presented to illustrate the themes/ﬁndings? Was each quotation identiﬁed? e.g. participant number | Yes, participant quotations are presented to illustrate the themes and each quotation is identified by participant number. | Findings, p. 5 |
| 30. Data and ﬁndings consistent | Was there consistency between the data presented and the ﬁndings? | The results are based on the interview statements | Findings, p. 5 |
| 31. Clarity of major themes | Were major themes clearly presented in the ﬁndings? | Yes, major themes are clearly identified. | Findings, p. 5 |
| 32. Clarity of minor themes | Is there a description of diverse cases or discussion of minor themes? | Yes, minor themes are clearly identified. | Findings, p. 5 |

**References**

1. Ehrenzeller S, Durovic A, Kuehl R, Martinez AE, Bielser M, Battegay M, et al. A qualitative study on safety perception among healthcare workers of a tertiary academic care center during the SARS-CoV-2 pandemic. Antimicrob Resist Infect Control. 2022;11(1):30.

2. Sandelowski M. Whatever happened to qualitative description? Res Nurs Health. 2000;23(4):334-40.

3. Schreier M. Qualitative content analysis in practice: Sage publications; 2012.

4. Tong A, Sainsbury P, Craig J. Consolidated criteria for reporting qualitative research (COREQ): a 32-item checklist for interviews and focus groups. International journal for quality in health care. 2007;19(6):349-57.

5. Kuckartz U. Analyzing Qualitative Data with MAXQDA : Text, Audio, and Video. 1st ed. 2019. ed. Rädiker S, editor. Cham: Springer International Publishing, Imprint: Springer; 2019.

6. Weiss RS. Learning from strangers: The art and method of qualitative interview studies: Simon and Schuster; 1995.

7. Padgett DK. Qualitative methods in social work research: Sage publications; 2016.

8. Lincoln YS, Lincoln YS, Guba EG, Guba EG. Naturalistic inquiry. Beverly Hills [etc: Sage Publications; 1985.

9. Ely M, Anzul M, Freidman T, Garner D, McCormack-Steinmetz A. Doing qualitative research: Circles within circles: Psychology Press; 1991.
